# Effects of morphological traits on body weight and analysis of growth-related genes of *Parabramis pekinensis* at different ages

**DOI:** 10.1186/s40850-023-00174-9

**Published:** 2023-08-24

**Authors:** Wentao Xu, Yaming Feng, Zhengyan Gu, Shuanglin Zhang, Zhijing Yang, Ye Xu, Hailong Gu

**Affiliations:** Taizhou Institute of Agricultural Science, Jiangsu Academy of Agricultural Sciences, Taizhou, 225300 China

**Keywords:** *Parabramis pekinensis*, Morphological traits, Body weight, Correlation analysis, GH-IGF-1 signaling pathway, TOR signaling pathway

## Abstract

*Parabramis pekinensis* was treated as research object in order to investigate the correlation between morphological traits and body weight. We measured 9 morphological indexes including total length (*X*_*1*_), body length (*X*_*2*_), body height (*X*_*3*_), head length (*X*_*4*_), snout length (*X*_*5*_), eye diameter (*X*_*6*_), eye distance (*X*_*7*_), caudal stalk length (*X*_*8*_) and caudal stalk height (*X*_*9*_). The principal morphological traits affecting body weight were screened out and the regression equation was established. The regression equation of Y1 (age 1 group) shape character (*X*) and weight (*Y*) was *Y* = − 169.183 + 32.544* × *_3_ + 10.263* × *_4_ + 15.655* × *_7_. The regression equation of Y2 (age 2 group) shape character (*X*) and weight (*Y*) was *Y* = − 694.082 + 7.725* × *_1_ + 72.822* × *_3_ + 77.023* × *_6_, the regression equation of Y3 (age 3 group) shape character (*X*) and weight (*Y*) was *Y* = − 1161.512 + 26.062* × *_1_ + 22.319* × *_2_- 107.218* × *_5_ + 83.901* × *_7_. Gene expression was consistent with these conclusions. TOR signaling pathway expression raised in Y1 then width increased. And GH-IGF-1 signaling pathway expression raised in Y2 then the length increased. In conclusion, the paper could prove that *P. pekinensis* showed a growth trend, which was increasing width first and length later. In some sense, the study not only enriched the basic biological data of *P. pekinensis*, but also provided waiting morphological traits for selective breeding of *P. pekinensis* artificial breeding in future.

## Introduction

*Parabramis pekinensis*, also called White bream or Beijing white bream, belongs to the *Cypriniformes*, *Cyprinidae*, *Culterinae*, *Parabramis*. It was a plentiful fish species that lived in Heilongjiang, Yangtze, Pearl River and other major water systems. But little studies were reported it. With the continuous improvement of people’s living standard, high-quality fish species such as *P. pekinensis* have captured an increasing amount of attention, and became progressively popular in the market of the Yangtze River Delta. Because of its fresh meat, rich protein and fat content, *P. pekinensis* was favored by consumers, with broad market prospect and desirable industry development momentum. However, *P. pekinensis* resources have been seriously threatened due to overfishing [1], damaged habitats and environmental adverse effects, which lead to reduced quantity, smaller body size, slower growth rate and weaker resistance to environmental changes. In order to reduce the persecution of *P. pekinensis* germplasm resources, researchers began to study the artificial propagation techniques and variety breeding in the hope of obtaining cultivars with excellent traits for cultivation and promoting. At present, the work of artificial breeding by catch wild *P. pekinensis* has reached the third generation. The slow growth rate and unstable breeding benefits were still difficult to overcome. As a result, it was urgent that we need to select and breed fast-growing species with better morphological traits to increase production and income for the aquaculture industry [2, 3].

Body weight, as a crucial growth index of fish, was also an important indicator for judging the efficacy of fish breeding. It was reported that the growth traits of fish were bound up with body weight [4, 5]. Therefore, it was significant for enriching the research on fish genetics and breeding by using statistical analysis methods to regression equations and explore the correlation between fish growth traits and body weight [6].

GH and IGF-1 were well known in aquaculture. In previous study, *Oncorhynchus keta* transgenic to GH gene had faster growth rate than control group [7]. In addition, other studies have shown GH could promote growth by injection and food supplementation [8, 9]. GH also could induce expression of liver IGF-1. Similar to GH, IGF-1 was also able to promote growth [10]. For example, increasing IGF-1 concentrations in tissues and serum of *Oreochromis niloticus*, *Oreochromis mossambicus*, *Oncorhynchus keta* and *Cirrhinus molitorella* promoted growth of the fish organism [11–13]. These studies confirmed that IGF-1 was a growth promoter for fish. But GH and IGF-1 promoted growth reflected in morphological parameters still unknown. It was found that the expression of TOR pathway differed at different growth stages. Because it could affect protein synthesis, which brought varying degrees of impact on growth [14]. Some studies have shown that when amino acids were abundant, TOR senses amino acid levels and was activated to enhance protein synthesis by affecting the downstream core genes AKT, S6, and 4EBBP1 [15, 16].

In this study, we used correlation analysis, path analysis and multiple regression analysis to investigate the growth traits associated with the body weight of *P. pekinensis* at different ages. Combined with regression equation and growth-related mRNA expression, we learnt to *P. pekinensis* growth pattern. And we hoped to established the corresponding to enrich the data for *P. pekinensis* breeding research.

## Methods

### Materials

**Fig. 1:**
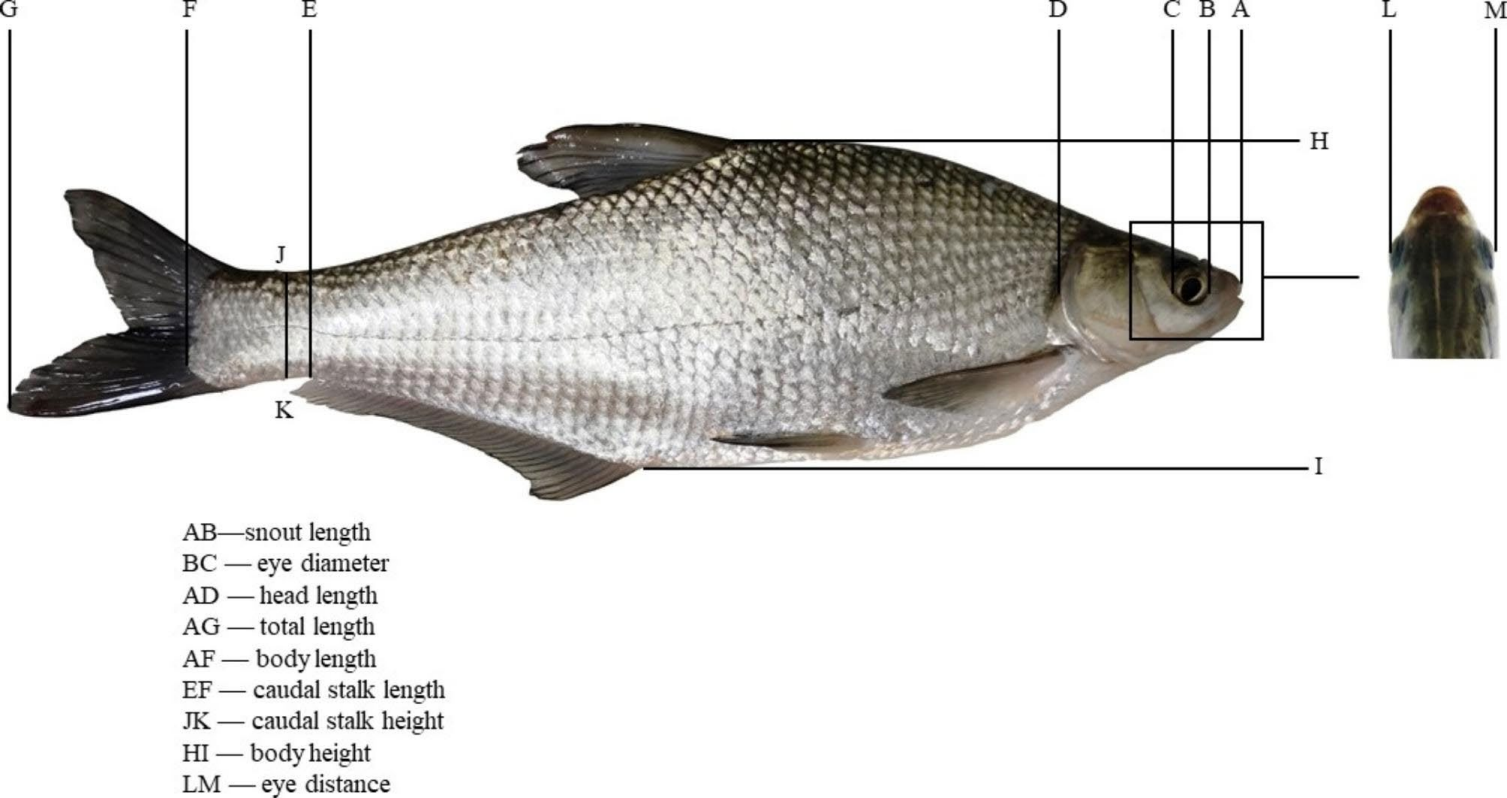
Morphometric traits of *P. pekinensis*

Three different years old *P. pekinensis* (Y1, Y2 and Y3 represented respectively the age of 1, 2 and 3) were randomly selected from Binjiang Aquaculture Breeding Farm in Jingjiang City, Jiangsu Province on September 26, 2022. The number of them were 48, 53 and 54 respectively. After anesthesia with 40 mg/L MS-222, 9 morphological indexes including total length (*X*_*1*_), body length (*X*_*2*_), body height (*X*_*3*_), head length (*X*_*4*_), snout length (*X*_*5*_), eye diameter (*X*_*6*_), eye distance (*X*_*7*_), caudal stalk length (*X*_*8*_) and caudal stalk height (*X*_*9*_) were measured by vernier caliper, accurate to 0.01 cm [17, 18]. The body weight (*Y*) was weighed with an electronic balance, accurate to 0.01 g. Specific parts of morphological traits as Fig. 1.

### Gene expression

After the breeding experiment, the fishes were all starved for 24 h. Then the fishes were dissected to obtain liver samples, which were stored in cryopreservation tubes for gene expression analysis. The related genes mRNA levels of protein synthesis were determined by qRT-PCR analysis, which was referred to Liang [19]. First, RNA extractionwas operated by the Bacteria RNA Extraction Kit (Vazyme BiotechCo., Ltd, China). Second, the thermo Scientific NanoDrop- 2000 Spectrophotometers (Thermofisher Scientific, USA) was used for testing the quantity and quality of RNA (Keep the OD260nm/OD280nm value at 1.8 ~ 2.0). Third, cDNA was synthesized by reverse transcription using Vazyme’s HiScripte ll 1st Strand cDNA Synthesis Kit (+ gDNA wiper) and then stored at -20 °C. Finally, qRT-PCR was analyzed using a CFX96 Touch Real-Time PCR Detection System (Bio-Rad, USA). The reaction system was showed in Table 1.

**Table 1:** Reaction system

Reaction system	
2×Taq Pro Universal SYBR qPCR Master Mix	10.0 µl
Upstream primer	0.4 µl
Downstream primer	0.4 µl
cDNA	1 µl
ddH2O	up to 20 µl

The reaction was performed as follows: Pre-denaturation at 95 °C for 30s; Cyclic denaturation at 95 °C for 10s, annealing and denaturation at 60 °C for 30s with 40 cycles; Melting reaction was 95 °C for 15s, 60 °C for 60s, then 95 °C for 10s, and read the plate for 30s. The primers for the analysis of mRNA levels were shown in Table 2. The Glyceraldehyde-3-phosphate dehydrogenase (GAPDH) was selected as internal reference gene, and the levels of mRNA were calculated from the standard curve, normalized against GAPDH and quantified using a relative standard curve method [20].

**Table 2:** Primers used for quantitative RT-PCR (qPCR)

Target genes	Sources	primer (5′-3′)
GAPDH^a^	Y09455.1	F: ACTGTCACTCCTCCATCTT
		R: CACGGTTGCTGTATCCAA
EF-1α^b^	AF467776.1	F: TCATTGGCCATGTCGACTCC
		R: ACGTAGTACTTGGCGGTCTC
TOR^c^	JN850959.1	F: TCTCCCTGTCCAGAGGCAATAA
		R: CAGTCAGCGGGTAGATCAAAGC
AKT^d^	MG516906.1	F: AGCGCACCTTCCATGTAGAC
		R: GGCTATTTGCCACTTGCTGG
S6^e^	XP_010747297.3	F: GTAATGCAAAGGACACGGCG
		R: GTTCCCCACCGCTCAGATAC
4EBP1^f^	NM_004095.4	F: AGCAGGAACTTTCGGTCATA
		R: GTCAATGGGCAGTCAGAAGA
GH^g^	KR269816.1	F: GACGGAGGAGCAGCGACA
		R: ACAGGGACCGACTGGGGA
IGF-1^h^	AY776159.1	F: TATTTCAGTAAACCAACAGGCTATG
		R: TGAATGACTATGTCCAGGTAAAGG

a: GAPDH, glyceraldehyde-3-phosphate dehydrogenase; b: EF-1α, elongation factors 1 alpha; c: TOR, target of rapamycin; d: AKT, protein kinase B; e: S6, ribosomal protein S6; f: 4EBP1, eukaryotic initiation factor 4E binding protein 1; g: GH, growth hormone; h: IGF-1, insulin-like growth factor-1

### Statistical analysis method

The data of 3 dissimilar ages and 10 diverse traits were sorted out by Excel software. Referring to the analysis method of Du [21], SPSS 22.0 software was also used for analyzing data by one-way analysis of variance (ANOVA) and Tukey’s multiple comparisons test. Path analysis and determination coefficient calculation refer to the method of Song [22]. In addition, all the analyzed data were presented as the means with S.E.M. *P* < 0.05, which was considered to be a significant difference.

## Results

### Statistics of phenotypic parameters and normal distribution test (body weight) of *P. pekinensis*

*P.pekinensis* phenotypic traits parameters of diverse ages were shown in Table 3. The coefficient of variation on 1 age body weight (Y) was the highest, which was 29.76%, and the head length (X4) was the lowest, which was 7.93%. The coefficient of variation on 2 ages body weight (Y) was the highest, which was 11.73%, and body length (X2) was the lowest, which was 4.24%. The coefficient of variation on 3 ages eye distance (X7) was the highest, which was 12.54%, and body length (X2) was the lowest, which was 3.99%.

**Table 3:** Parameter statistics of phenotypic traits in *P. pekinensis*

age	parameter	*Y*/g	*X*_1_/cm	*X*_2_/cm	*X*_3_/cm	*X*_4_/cm	*X*_5_/cm	*X*_6_/cm	*X*_7_/cm	*X*_8_/cm	*X*_9_/cm
Y1	mean	80.54	19.82	14.66	5.93	3.39	0.96	0.97	1.40	2.08	1.77
	SD	23.97	1.96	1.68	0.58	0.27	0.17	0.15	0.17	0.21	0.19
	CV/%	29.76	9.86	11.44	9.75	7.93	17.49	15.77	12.24	10.20	10.76
Y2	mean	474.27	36.11	27.25	10.64	5.81	1.49	1.50	2.41	3.80	3.38
	SD	55.64	1.60	1.28	0.62	0.28	0.11	0.10	0.24	0.31	0.21
	CV/%	11.73	4.43	4.70	5.80	4.89	7.56	6.51	9.96	8.07	6.22
Y3	mean	699.55	40.29	33.78	12.31	6.48	1.64	1.62	2.78	4.15	3.67
	SD	82.60	1.61	1.35	0.62	0.29	0.18	0.15	0.35	0.32	0.17
	CV/%	11.81	4.00	3.99	5.00	4.42	11.12	9.54	12.54	7.80	4.76

Note: Y. body weight; X_1_. total length; X_2_. body length; X_3_. body high; X_4_. head length; X_5_. snout length; X_6_. eye diameter; X_7_. eye distance; X_8_. caudal stalk length; X_9_. caudal stalk height

The normal distribution test results of body weight were shown in Table 4. Kolmogorov-Smirnov and Shapiro-Wilk and other methods were adopted to test *P. pekinensis* weight’s normal distribution, and the obtained significance levels were all greater than 0.05, which indicated that body weight of three groups obeys normal distribution. It suggested that the dates satisfied the condition of regression analysis.

**Table 4:** The normal distribution test of body weight in *P. pekinensis*

	Kolmogorov-Smirnova			Shapiro-Wilk		
age	statistic	d*f*	*P*	statistic	d*f*	*P*
Y1	0.109	48	0.200	0.948	48	0.320
Y2	0.113	53	0.088	0.968	53	0.160
Y3	0.105	54	0.200	0.970	54	0.198

### Correlation analysis of phenotypic traits in *P. pekinensis* at different ages

The correlation coefficients with *P. pekinensis* 45 pairs of dissimilar ages were shown in Table 5. In Y1, the results showed that there was significant association between the phenotypic traits of 45 pairs (*P* < 0.05), all 9 morphological traits were remarkably correlated with body weight (*P* < 0.05); In Y2, there was a remarkable correlation between 39 pairs of phenotypic traits (*P* < 0.05), except for tail stalk length (*X*_*8*_), there was no significant correlation with body weight (*P* > 0.05), the other 8 pairs of phenotypic traits were noticeably correlated with body weight (*P* < 0.01); In Y3, There was a striking association between the phenotypic traits of 43 pairs (*P* < 0.05), except for tail stalk length (*X*_*8*_), there was no significant correlation with body weight (*P* > 0.05), the other 8 pairs of phenotypic traits were markedly correlated with body weight (*P* < 0.01). As indicated by comprehensive analysis, the correlation of shape traits was consistent to some extent between juvenile and adult, except tail stalk length (*X*_*8*_), the other traits had high correlation with body weight, which required further analysis.

**Table 5:** Correlation analysis of phenotypic traits in *P. pekinensis*

age	trait	*Y*	*X*_1_	*X*_2_	*X*_3_	*X*_4_	*X*_5_	*X*_6_	*X*_7_	*X*_8_
Y1	*X*_1_	0.953^**^								
	*X*_2_	0.904^**^	0.921^**^							
	*X*_3_	0.980^**^	0.939^**^	0.886^**^						
	*X*_4_	0.920^**^	0.917^**^	0.844^**^	0.957^**^					
	*X*_5_	0.897^**^	0.852^**^	0.801^**^	0.870^**^	0.845^**^				
	*X*_6_	0.914^**^	0.891^**^	0.854^**^	0.855^**^	0.821^**^	0.861^**^			
	*X*_7_	0.892^**^	0.925^**^	0.861^**^	0.863^**^	0.858^**^	0.780^**^	0.857^**^		
	*X*_8_	0.946^**^	0.956^**^	0.890^**^	0.936^**^	0.906^**^	0.795^**^	0.866^**^	0.897^**^	
	*X*_9_	0.870^**^	0.849^**^	0.799^**^	0.859^**^	0.832^**^	0.799^**^	0.843^**^	0.757^**^	0.830^**^
Y2	*X*_1_	0.728^**^								
	*X*_2_	0.759^**^	0.909^**^							
	*X*_3_	0.929^**^	0.673^**^	0.757^**^						
	*X*_4_	0.628^**^	0.653^**^	0.713^**^	0.562^**^					
	*X*_5_	0.626^**^	0.502^**^	0.551^**^	0.611^**^	0.638^**^				
	*X*_6_	0.553^**^	0.363^*^	0.473^*^	0.507^**^	0.539^**^	0.696^**^			
	*X*_7_	0.785^**^	0.562^**^	0.599^**^	0.781^**^	0.625^**^	0.659^**^	0.435^*^		
	*X*_8_	0.291	0.463^*^	0.541^**^	0.274	0.174	0.212	0.189	0.145	
	*X*_9_	0.822^**^	0.674^**^	0.723^**^	0.783^**^	0.563^**^	0.749^**^	0.551^**^	0.817^**^	0.387^*^
Y3	*X*_1_	0.939^**^								
	*X*_2_	0.925^**^	0.925^**^							
	*X*_3_	0.711^**^	0.690^**^	0.655^**^						
	*X*_4_	0.748^**^	0.773^**^	0.737^**^	0.489^*^					
	*X*_5_	0.749^**^	0.834^**^	0.680^**^	0.585^**^	0.675^**^				
	*X*_6_	0.785^**^	0.881^**^	0.775^**^	0.690^**^	0.718^**^	0.878^**^			
	*X*_7_	0.821^**^	0.823^**^	0.709^**^	0.594^**^	0.679^**^	0.886^**^	0.785^**^		
	*X*_8_	0.383^*^	0.423^*^	0.379^*^	0.262	0.241	0.322^*^	0.358^*^	0.368^*^	
	*X*_9_	0.812^**^	0.807^**^	0.744^**^	0.571^**^	0.728^**^	0.728^**^	0.730^**^	0.788^**^	0.387^*^

Note: ** indicates extremely significant effect (*P* < 0.01), * indicates significant effect (*P* < 0.05). Y. body weight; X_1_. total length; X_2_. body length; X_3_. body high; X_4_. head length; X_5_. snout length; X_6_. eye diameter; X_7_. eye distance; X_8_. caudal stalk length; X_9_. caudal stalk height

### Path analysis and determination coefficients of morphological traits on body weight in *P. pekinensis*

In accordance with results of correlation analysis, the path analysis was carried out on the shape character and body weight (Table 6). The direct effect of these three morphological traits on weight was body height (*X*_*3*_) > head length (*X*_*4*_) > eye distance (*X*_*7*_) in sequence, and the indirect effect was in the same order with direct effect. Morphological traits retained in Y2 were body height (*X*_*3*_), head length (*X*_*4*_) and caudal stalk height (*X*_*9*_). The direct effects of these morphological traits on body weight were body height (*X*_*4*_) > head length (*X*_*4*_) > caudal stalk height (*X*_*9*_), and the indirect effects were in the same order with direct effects. Morphological traits retained in Y3 were total length (*X*_*1*_), body length (*X*_*2*_), snout length (*X*_*5*_) and eye distance (*X*_*7*_). The order of direct effect on body weight was full length (*X*_*1*_) > body length (*X*_*2*_) > eye distance (*X*_*7*_) > kiss length (*X*_*5*_), and the order of indirect effect was total length (*X*_*1*_) > eye distance (*X*_*7*_) > body length (*X*_*2*_) > snout length (*X*_*5*_). The indirect effect of snout length was negatively correlated with body weight.

**Table 6:** Path analysis of morphological traits on body weight in *P. pekinensis*

age	trait	correlation coefficient	direct effect	indirect effect				
				X3	X4	X7		Σ
Y1	X3	0.986**	0.786	-	0.711	0.706		1.416
	X4	0.920**	0.114	0.103	-	0.094		0.197
	X7	0.892**	0.112	0.101	0.092	-		0.193
Y2				X3	X4	X9		
	X3	0.786**	0.724	-	0.536	0.340		0.876
	X4	0.937**	0.195	0.144	-	0.116		0.260
	X9	0.637**	0.116	0.054	0.069	-		0.123
Y3				X1	X2	X5	X7	
	X1	0.789**	0.508	-	0.470	0.424	0.418	1.312
	X2	0.775**	0.364	0.337	-	0.248	0.258	0.842
	X5	0.599**	-0.237	-0.198	-0.161	-	-0.210	-0.569
	X7	0.671**	0.354	0.291	0.251	0.314	-	0.856

Note: ** indicates extremely significant effect (*P* < 0.01). *Y*. body weight; *X*_1_. total length; *X*_3_. body high; *X*_4_. body height; *X*_5_. snout length; *X*_7_. eye distance; *X*_9_. caudal stalk height

The determining coefficient of shape traits on weight in diverse ages was shown in Table 7. The determining effect of traits on weight was dissimilar in diverse ages. In Y1, the determining effect of body height on weight was 0.618. Under the combined condition of 2 forms, the determining effect of body height (*X*_*3*_) and head length (*X*_*4*_) on weight was 0.162. Followed by body height (*X*_*3*_) and eye distance (*X*_*7*_) on weight was 0.158. Both of them were higher than combined determining effect of head length (*X*_*4*_) and eye distance (*X*_*7*_). In Y2, the determination effect of total length (*X*_*1*_) on weight was the most importance, which was 0.524, far exceeds of other two forms. Under the combined condition of 2 forms and traits, the determination effect of total length (*X*_*1*_) and height (*X*_*3*_) on weight was the highest, which was 0.209. In Y3, the total length (*X*_*1*_) had the most remarkable decisive effect on weight, which was 0.258, and snout length (*X*_*5*_) had the least determining effect on weight, which was 0.056. Under the combined condition of two forms and traits, the full length (*X*_*1*_) and body length (*X*_*2*_) had the most noticeable decisive effect on weight, which was 0.342.

**Table 7:** Determination coefficients of morphological traits on body weight in *P. pekinensis*

age	trait					
Y1		X3	X4	X7		Σ
	X3	0.618	-	-		0.984
	X4	0.162	0.013	-		
	X7	0.158	0.021	0.013		
Y2		X1	X3	X6		Σ
	X1	0.524	-	-		0.888
	X3	0.209	0.038	-		
	X6	0.079	0.024	0.013		
Y3		X1	X2	X5	X7	Σ
	X1	0.258	-	-	-	0.926
	X2	0.342	0.132	-	-	
	X5	-0.201	-0.117	0.056	-	
	X7	0.296	0.183	-0.149	0.125	

Note: *Y*. body weight; *X*_1_. total length; *X*_3_. body high; *X*_4_. body height; *X*_5_. snout length; *X*_7_. eye distance; *X*_9_. caudal stalk height

### Regression analysis of *P. pekinensis* morphological traits on weight

Taking the morphological traits retained by *P. pekinensis* in the trialling analysis of dissimilar ages as independent variables, the stepwise regression analysis method was adopted. New variables were introduced into the regression equation successively and new regression analysis models are built constantly until the optimal regression equation was obtained. The multiple correlation analysis results of dissimilar regression analysis models were shown in Table 8. With the introduction of new variables into the regression analysis model, the multiple correlation coefficient R of each age stage was gradually increasing. When morphological traits were completely introduced into the regression analysis, the multiple correlation coefficient R were 0.990, 0.951 and 0.963. The error probability P were 0.040, 0.038 and 0.019 separately, which have statistical significance. It indicated that the retained variable was the primary morphological character affecting body weight in this age stage.

**Table 8:** Multiple-correlation analysis of the morphometric traits to the body weight in *P. pekinensis* Note: Y. body weight; X_1_. total length; X_2_. body length; X_3_. body high; X_4_. head length; X_5_. snout length; X_6_. eye diameter; X_7_. eye distance

age	parameters introduced in the model	*R*	*R* ^2^	adjusted *R*^2^	standard estimate error	*P*
Y1	X3	0.986	0.972	0.972	4.084	0.00
	X3, X7	0.989	0.977	0.976	3.722	0.002
	X3, X4, X7	0.990	0.979	0.978	3.586	0.040
Y2	X3	0.937	0.900	0.876	24.873	0.000
	X3, X1	0.947	0.897	0.893	23.141	0.004
	X3, X1, X6	0.951	0.905	0.900	22.376	0.038
Y3	X1	0.939	0.882	0.880	28.921	0.000
	X1, X2	0.951	0.900	0.900	26.375	0.001
	X1, X2, X7	0.959	0.919	0.914	24.435	0.003
	X1, X2, X7, X5	0.963	0.928	0.922	23.316	0.019

In line with the results of the multiple correlation analysis, the significance test was carried out on the bias regression coefficient and regression constant of the regression analysis model which introduced all morphological traits (Table 9) (*P* < 0.05). By comparing the regression coefficients of distinct forms and traits in the regression analysis model, it was found that *P. pekinensis* shape and traits were influenced by dissimilar forms in diverse ages. In Y1, the regression coefficients of form traits were body height (*X*_*3*_), head length (*X*_*4*_) and eye distance (*X*_*7*_), which were arranged in a descending order. In Y2, the regression coefficient of form traits was body height (*X*_*3*_), total length (*X*_*1*_) and eye diameter (*X*_*6*_) in descending order. In Y3, form traits regression coefficient was total length (*X*_*1*_), body length (*X*_*2*_), eye distance (*X*_*7*_) and snout length (*X*_*5*_) in descending order. The result indicated that body height (*X*_*3*_) worked as the highest trait influence in Y1 and Y2. While total length (*X*_*1*_) worked as the highest trait in Y3, which corresponded with the results of path analysis and determination coefficient analysis. In accordance with the partial regression coefficient of distinct forms, the multiple regression equations of *P. pekinensis* form traits and weight in diverse ages were established.

**Table 9:** Significance test of partial regression coefficient and regression constant

age	model	partial regression coefficient		regression coefficient	t	*P*
		B	SE			
Y1	(constant)	-169.183	7.166		-23.608	0.000
	X3	32.544	2.313	0.786	14.072	0.000
	X7	15.655	6.491	0.112	2.412	0.020
	X4	10.263	4.845	0.114	2.118	0.040
Y2	(constant)	-694.082	65.101		-10.662	0.000
	X3	72.822	7.169	0.724	10.158	0.000
	X1	7.725	2.577	0.195	2.998	0.004
	X6	77.023	36.123	0.116	2.132	0.038
Y3	(constant)	-1161.512	111.002		-10.464	0.000
	X1	26.062	7.666	0.508	3.4	0.001
	X2	22.319	6.864	0.364	3.252	0.002
	X7	83.901	20.809	0.354	4.032	0.000
	X5	-107.218	44.098	-0.237	-2.431	0.019

Note: Y. body weight; X_1_. total length; X_2_. body length; X_3_. body high; X_4_. head length; X_5_. snout length; X_6_. eye diameter; X_7_. eye distance

Age 1: *Y*=-169.183 + 32.544* × *_3_ + 10.263* × *_4_ + 15.655* × *_7_

Age 2: *Y*=-694.082 + 7.725* × *_1_ + 72.822* × *_3_ + 77.023* × *_6_

Age 3: *Y*=-1161.512 + 26.062* × *_1_ + 22.319* × *_2_-107.218* × *_5_ + 83.901* × *_7_

In formula, *Y* represents weight, *X*_*1*_ represents total length, *X*_*2*_ represents body length, *X*_*3*_ represents body height, *X*_*4*_ represents head length, *X*_*5*_ represents snout length, *X*_*7*_ represents eyes distance, and *X*_*9*_ represents tail stalk height.

### Growth gene expression analysis

**Fig. 2:**
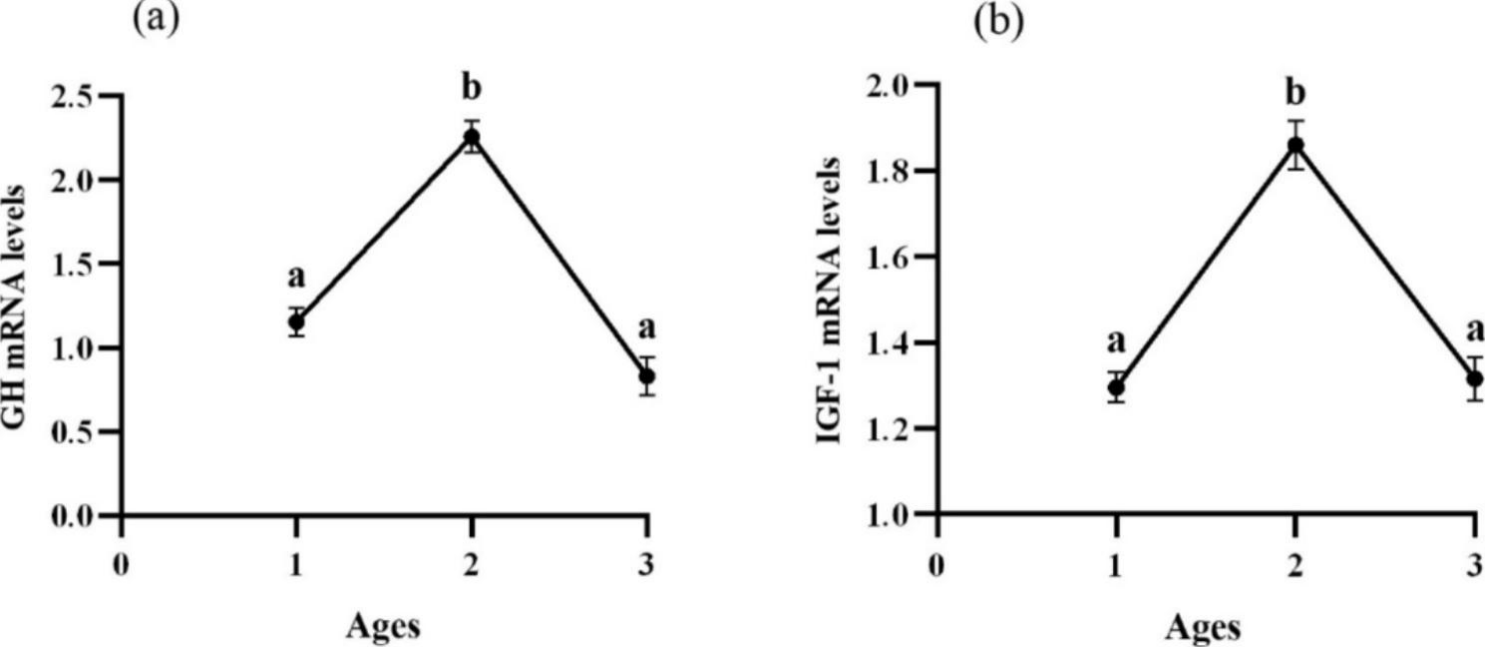
The relative expressions of GH-IGF-1 axis in liver of *P. pekinensis* with different ages. Data are expressed as means with S.E.M.; value with different superscripts are significantly different (P < 0:05)

**Fig. 3:**
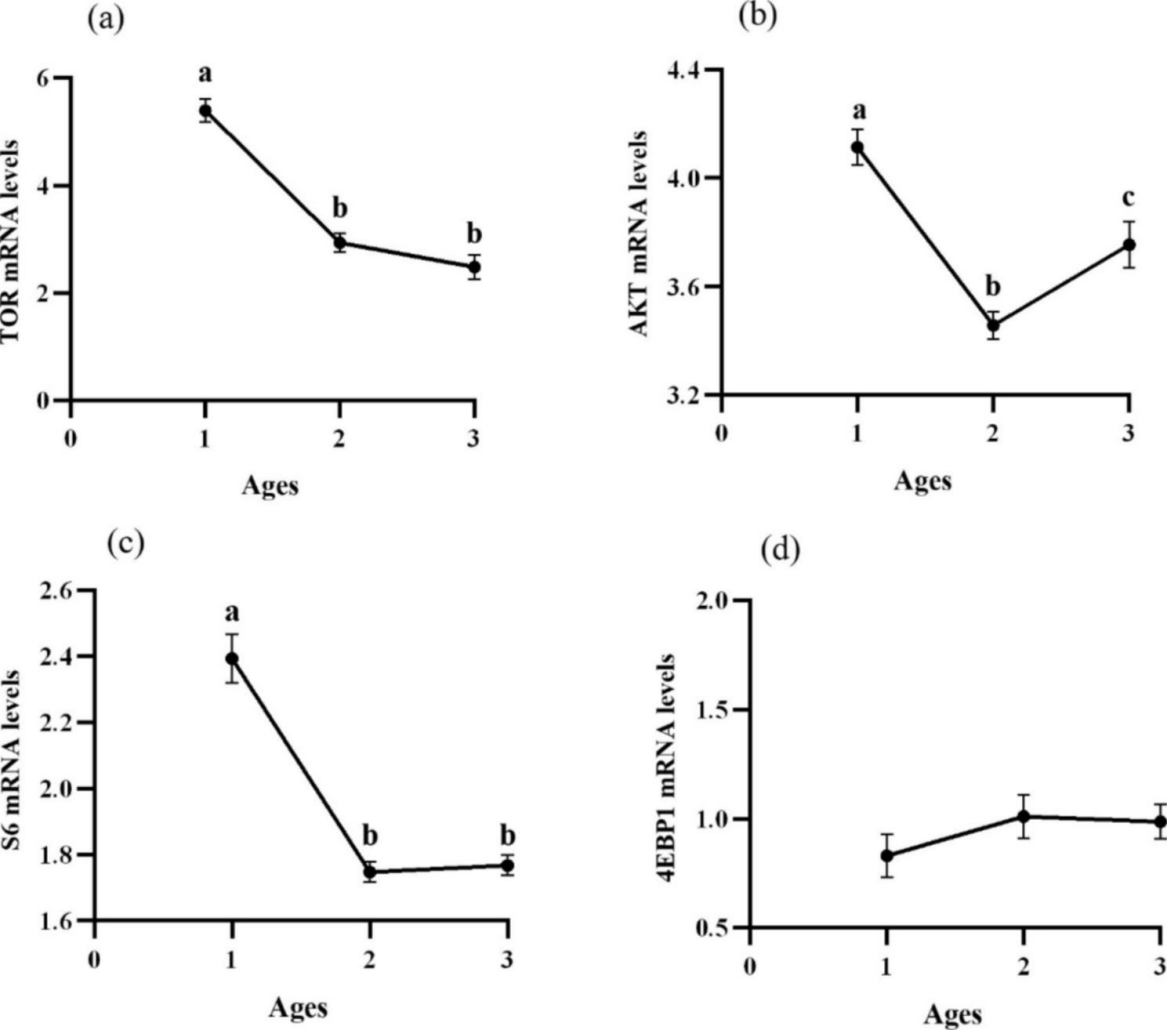
The relative expressions of TOR signaling pathway in liver of *P. pekinensis* with different ages. Data are expressed as means with S.E.M.; value with different superscripts are significantly different (P < 0:05)

Y2 showed significantly increased mRNA levels of growth hormone (GH) and insulin-like growth factor-1 (IGF-1) (P < 0.05) (Fig. 2(a) and 2(b)). Furthermore, target of rapamycin (TOR) and ribosomal protein S6 (S6) expressed the same trend which Y1 showed significantly higher mRNA levels (P < 0.05) (Fig. 3(a) and 3(c)). AKT performed similar trend with TOR and S6, but it showed significantly difference between Y2 and Y3 (P < 0.05) (Fig. 3(b)). However, no significant effects on the mRNA levels of eukaryotic initiation factor 4E binding protein 1 (4EBP1) were found in liver of *P. pekinensis* with different ages (P > 0.05) (Fig. 3(d)).

## Discussion

### Correlation between morphological traits and body weight

In the study of fish biology, body weight was often considered as the uppermost indicator to measure the body size of fish, followed by body length [23, 24]. As revealed by comparing the statistical results of diverse ages, the average weight of *P. pekinensis* grows from 80.54 to 699.55 g from 1 to 3 ages, with growth rate of 769%, which indicated that *P. pekinensis* grew tremendously fast. With the growth of age, the coefficient of variation on *P. pekinensis* weight and body length lessens progressively. The coefficient of variation on weight decreases from 29.76 to 11.81% and body length decreases from 11.44 to 3.99%. As indicated by other experimental findings, the individual of *P. pekinensis* differs immensely from adult stage, in which weight and body length could be affected by environmental factors. In juvenile stage, the growth of fish body was affected by the environment lightly and often correlated with fish body; while in adult stage, the influence of environmental factors on fish growth was enhanced, and the fish population showed homogeneity. In breeding work, juvenile stage had more selective potential than adult stage. If environmental difference was not significant, further analysis was needed.

Correlation analysis could define correlation between multiple things, help find more closely related variables, eliminate irrelevant variables, and make statistical judgments [25]. Other study severally explored the association between morphological traits and body weight of male and female *Scatophagus argus* [26]. The study showed that correlation coefficient was affected by sex, and it was dissimilar between morphological traits and body weight. And another study showed the correlation between morphological traits and body weight of 1^+^~3^+^ ages *Thymallus arcticus*, and showed morphological traits correlated with body weight are dissimilar in different ages. The number of traits decreased with the increased of ages [27]. Similarly, Liu studied the association between body weight and morphological traits of *Larimichthys crocea* in 13 and 20 months [28]. It indicated that the correlation coefficients between body weight and morphological traits were dissimilar at different ages. Similar results have been found in *Macrobrachium rosenbergii* [29] and *Phoxinus lagowskii* [30]. As the *P. pekinensis* was growing with the same speed [31], there was little difference in morphological traits between different ages. With the ages increased, the correlation coefficient progressively lessens became smaller, showing that the growth rate was faster in juvenile stage. In this study, the correlation coefficients between Y1 morphological traits and weight from high to low were body height (*X*_*3*_), total length (*X*_*1*_), tail stalk length (*X*_*8*_), head length (*X*_*4*_), eye diameter (*X*_*6*_) and body length (*X*_*2*_) correlation coefficient > 0.90), while the Y2 only body height (*X*_*3*_) had correlation coefficient higher than 0.9. The correlation coefficient total length (*X*_*1*_) and body length (*X*_*2*_) were higher than 0.9 in Y3 and total length (*X*_*1*_) was higher than body length (*X*_*2*_). It could be seen that the correlation between different traits and body weight differs tremendously, and the correlation between same morphological traits and body weight was also dissimilar at diverse growth stages. As persuasively illustrated in the research completed by Huang [32], the trait which could directly exert the most conspicuous influence on body weight of 2-month-old yellow croaker was the total length, while body height was the key trait affected body weight of 18-month-old yellow croaker. Other study held a standpoint that body length was the chief trait affecting body quality of Masu salmon at 6 months, while body height, caudal stalk height and head height were also the trait affecting body quality of Masu salmon at 18 months [33]. As a consequence, it was imperative to investigate the effects of morphological traits on body weight in three age groups. In this experiment, the correlation coefficients including body length (*X*_*2*_) and body height (*X*_*3*_) showed high trends with body weight in three age groups, indicating that body length, body height and body weight maintained the highest level of correlation. The conclusion coincided with the biological characteristics of *P. pekinensis* [34], because *P. pekinensis* paid attention to both lateral and vertical growth during the growth stage.

### Main morphological traits affecting body weight

Correlation analysis could obtain simple association between morphological traits and body weight. Path analysis was adopted in the study to probe deep into the direct and indirect effects between traits [35], so as to obtain more reliable data support and basis. In the study of *Ruditapes philippinarum*, Wang found that shell length, shell height, shell width and shell thickness were the main factors affecting live weight, while gonads were a dominant factor affecting soft weight [36]. Chen [37] analyzed the effects of morphological traits on body weight of flounder at distinct periods, and found that the length from the base of pelvic fin to the terminal of dorsal fin had the highest diameter coefficient on body weight at 8 months and 14 months, which were 0.439 (8 months) and 0.752 (14 months). In the study, the direct path coefficients of 3 morphological traits on body weight reached extremely substantial level in 1 and Y2, while 4 traits in Y3. In 1 and 2 ages growth stages, the diameter coefficient of body height (*X*_*3*_) on body weight was the highes. Then it was followed by head length (*X*_*4*_). In 3 ages growth stage, the maximum diameter coefficients of total length (*X*_*1*_) and body height (*X*_*3*_) on body weight were 0.789 and 0.775. The results of direct effects coincided with above conclusions. In 1 and 2 ages, body height (*X*_*3*_) had the largest direct effect on body weight. The direct effect of total length (*X*_*1*_) and body length (*X*_*2*_) on body weight in Y3 were 0.508 and 0.364. As a consequence, it was essential to select diverse morphological traits as breeding materials at distinct ages. As for *P. pekinensis*, body height was the selection traits before 2 years old, and selecting total length and body length after 2 years old were suitable.

Multiple regression analysis was conducted on the basis of path analysis, which could reinforce the reliability of regression equation [38]. The determining coefficient could be employed to judge whether retained shape traits were the dominant influencing factor of body weight in the path analysis. Haiweis [39] believed that the sum of determining coefficient Σ ≥ 0.850 indicated that retained shape traits were the predominant influencing factor of weight. The total determination coefficients of three different groups were 0.984, 0.888 and 0.926, which indicated that selected morphological traits of 3 ages were the principal morphological traits affecting body weight. The effects of morphological traits on fish body weight were dissimilar at different ages. This phenomenon has been reported in *Nibea albiflora* [40], *Ctenopharyngodon idella* [41] and *Cynoglossus semilaevis* [42]. As demonstrated by correlation analysis, path analysis and multiple regression analysis, in Y1, the predominant morphological traits affecting body weight were body height (*X*_*3*_) and head length (*X*_*4*_). In Y2, the primary morphological traits affecting body weight were total length (*X*_*1*_) and body height (*X*_*3*_). Finally, in Y3, the chief morphological traits affecting body weight were total length (*X*_*1*_) and body length (*X*_*2*_).

### TOR and GH-IGF-1 signaling pathway analysis

**Fig. 4:**
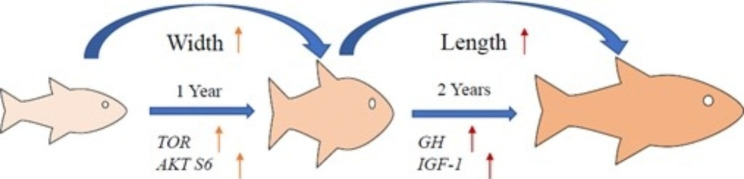
Diagram of different ages growth patterns

The GH-IGF-1 axis had an important role in regulating somatic growth [43]. It has been shown that the expression of GH, IGF-1 and TOR signaling pathways ccould represent not only growth level, but also the increase in body weight and production [44]. In the study, the mRNA levels of GH and IGF-1 were significantly upregulated in Y2, which partly represented that *P. pekinensis* vertical grew rapidly in 2 years old. TOR signaling system could sense amino acid abundance [45]. In this study, TOR, S6 and AKT gene expression significantly increased in Y1. And AKT gene expression showed difference in 3 years. Previous studies have confirmed that TOR signaling pathway and protein synthesis could affect growth performance in grass carp and blunt snout bream, which affected the downstream gene expression to regulate protein synthesis [46, 47]. However, the expressions of TOR signaling pathway were the opposite trend with the mRNA levels of GH and IGF-1. The conclusions suggested that *P. pekinensis* growth could be affected by expression of these genes, but the degree of gene expression varies from different ages, and the growth pattern was consistent with above study of morphology and shape. During the whole process, the width was increased at age 0 ~ 1, while the length was increased after at age 1 ~ 3 (Fig. 4). The result might be related to absorbed nutrients and gonad development. Relevant research needed further demonstrated.

## Conclusions

The traits affecting body weight varied in different ages. Individual differences in juvenile *P. pekinensis* were greater than adult stage, and it became stable in growth progress. Correlation coefficients also differed in three ages, but body length and body height always maintained a high correlation with body weight. According to path analysis and multiple regression analysis, main morphological traits affecting body weight in different ages were diverse. Y1 was body height and head length; Y2 was total length and body height; Y3 was total length and body length. Gene expression was consistent with these conclusions. TOR signaling pathway expression raised in Y1 then width increased. And GH-IGF-1 signaling pathway expression raised in Y2 then the length increased. In conclusion, the paper could prove that *P. pekinensis* showed a growth trend, which was increasing width first and length later. The study provided morphological traits for selective breeding of *P. pekinensis* artificial breeding in future.

## Data Availability

The data that support the findings of this study are available from the corresponding author upon reasonable request.
